# Light-Induced Fos Expression in Intrinsically Photosensitive Retinal Ganglion Cells in Melanopsin Knockout (Opn4^−/−^) Mice

**DOI:** 10.1371/journal.pone.0004984

**Published:** 2009-03-25

**Authors:** Gary E. Pickard, Scott B. Baver, Malcolm D. Ogilvie, Patricia J. Sollars

**Affiliations:** Division of Neuroscience, Department of Biomedical Sciences, Colorado State University, Fort Collins, Colorado, United States of America; Vrije Universiteit Amsterdam, Netherlands

## Abstract

Retinal ganglion cells that express the photopigment melanopsin are intrinsically photosensitive (ipRGCs) and exhibit robust synaptically driven ON-responses to light, yet they will continue to depolarize in response to light when all synaptic input from rod and cone photoreceptors is removed. The light-evoked increase in firing of classical ganglion cells is determined by synaptic input from ON-bipolar cells in the proximal sublamina of the inner plexiform layer. OFF-bipolar cells synapse with ganglion cell dendrites in the distal sublamina of the inner plexiform layer. Of the several types of ipRGC that have been described, M1 ipRGCs send dendrites exclusively into the OFF region of the inner plexiform layer where they stratify near the border of the inner nuclear layer. We tested whether M1 ipRGCs with dendrites restricted to the OFF sublamina of the inner plexiform layer receive synaptic ON-bipolar input by examining light-induced gene expression *in vivo* using melanopsin knockout mice. Mice in which both copies of the melanopsin gene (*opn4*) have been replaced with the *tau-lacZ* gene (homozygous *tau-lacZ^+/+^* knockin mice) are melanopsin knockouts (*opn4^−/−^*) but M1 ipRGCs are specifically identified by their expression of β-galactosidase. Approximately 60% of M1 ipRGCs in *Opn4^−/−^* mice exposed to 3 hrs of light expressed c-Fos; no β-galactosidase-positive RGCs expressed c-Fos in the dark. Intraocular application of L-AP4, a compound which blocks transmission of visual signals between photoreceptors and ON-bipolar cells significantly reduced light-evoked c-Fos expression in M1 ipRGCs compared to saline injected eyes (66% saline vs 27% L-AP4). The results are the first description of a light-evoked response in an ipRGC lacking melanopsin and provide *in vivo* confirmation of previous *in vitro* observations illustrating an unusual circuit in the retina in which ganglion cells sending dendrites to the OFF sublamina of the inner plexiform layer receive excitatory synaptic input from ON-bipolar cells.

## Introduction

Retinal ganglion cells that express the photopigment melanopsin are intrinsically photosensitive (ipRGCs) and depolarize in response to light in the absence of all synaptic input from rod and cone photoreceptors [1]–[4]. Dendrites of ipRGCs in the inner plexiform layer (IPL) are postsynaptic to bipolar and amacrine cells [5]–[7] and ipRGCs receive robust synaptically driven excitatory input [8]–[12]. In the mouse, several types of ipRGC have been described; M1 ipRGCs have dendrites that stratify in the distal sublamina of the IPL, M2 ipRGCs send dendrites into the proximal sublamina of the IPL, and a third type of ipRGC (M3) is bistratified with dendrites in both the proximal and distal IPL [7], [11]–[14].

Signals generated in rod and/or cone photoreceptors are conveyed through the retina via two functional circuits. Neurons belonging to the ON channel are depolarized by the onset of light; members of the OFF channel depolarize when light is turned off [15]. ON and OFF functional channels are created as the result of the differential expression of glutamate receptors on the dendrites of bipolar cells. ON bipolar cells use a metabotropic receptor, mGluR6, which functionally inverts the light-activated hyperpolarizations of photoreceptors into a depolarization [16]–[19]. OFF bipolar cells express ionotropic AMPA/kainate receptors [20]. The separate ON and OFF channels established at the photoreceptor-to-bipolar synapse are retained by conventional ganglion cells by the restriction of their dendrites to either the upper (OFF) or lower (ON) stratum of the IPL to receive synapses from OFF and ON bipolar cells, respectively [21].

It has previously been reported that ipRGCs do not maintain this strict anatomically determined differentiation between ON and OFF signaling observed in conventional ganglion cells. In the primate, two types of monostratified ipRGC have been described that send dendrites to either the inner or the outer stratum of the IPL and both types generate sustained ON responses to light *in vitro* [8]. It has also been suggested that OFF-stratifying ipRGCs in the rat receive ON-bipolar cell input as determined from *in vitro* recordings of ipRGCs that project to the hypothalamus [10], although the evidence in this latter case is less compelling because the morphology of the recorded cells was not directly examined. Nonetheless, it would appear that unless the responses recorded *in vitro* do not reflect the physiological responses of these cells to light *in vivo*, the M1 ipRGC responses to rod/cone driven synaptic input do not conform to the established ON-OFF functional stratification scheme of the IPL in the vertebrate retina.

To further explore the rod/cone driven synaptic response of M1 ipRGCs we examined light-induced expression of the protein product of the immediate early gene *c-fos in vivo* using a knockin mouse model. Fos expression has been widely used to map functional neuronal circuits in the CNS including the retina. It is possible to map functional circuits in the retina using light-stimulated induction of *c-fos* because Fos is virtually undetectable in the inner retina in the absence of light. Fos expression following light stimulation has been described in the retinal ganglion cell layer and in the inner nuclear layer (INL) of several species and in animals possessing deficits in specific retinal circuits [22]–[28].

To test directly whether ipRGCs with dendrites terminating in the OFF sublamina of the IPL receive ON-bipolar signals, we examined light-induced c-Fos expression in a mouse model in which the M1 ipRGCs are specifically identified and in which the intrinsic response to light, mediated by melanopsin, has been eliminated. Mice in which both copies of the melanopsin *opn4* gene have been replaced with the *tau-lacZ* gene (*tau-lacZ^+/+^* mice) are completely devoid of melanopsin (melanopsin knockouts, opn4^−/−^) [2], [13], [14], [29]. The M1 ipRGCs in *tau-lacZ^+/+^* mice are specifically identified by their expression of the β-galactosidase enzyme fusion protein; other ipRGCs apparently do not express enough β-galactosidase to be detected in the *tau-lacZ* mouse [13], [14]. Thus, light-induced Fos expression in β-galactosidase positive ipRGCs in the *tau-lacZ^+/+^* animal must by implication be synaptically driven. If synaptically driven excitatory input from ON-bipolar cells mediates light-induced Fos expression, then this circuit should be amenable to pharmacological blockade by L-AP4, an agonist of the mGluR6 receptor which blocks transmission of visual signals between photoreceptors and ON-bipolar cells [30].

## Results

### Light-induced Fos Expression in ipRGCs

**Figure 1:**
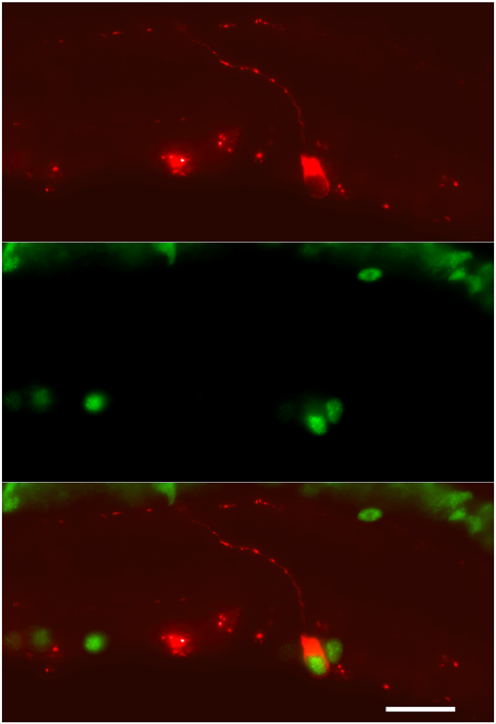
Light-induced c-Fos expression in an ipRGC lacking melanopsin. (upper panel) An M1 ipRGC in the retina of a *tau-lacZ^+/+^* mouse identified by β-galactosidase immunostaining. Note the dendrite emanating from this ipRGC extends to the outer aspect of the IPL, bifurcates and stratifies along the border of the INL, contributing to the definition of this ganglion cell as an M1 ipRGC. (middle panel) Same section as in upper panel illustrating light-induced Fos expression in the nuclei of neurons in the ganglion cell layer and INL. (bottom panel) Merged image illustrating light-induced Fos expression in an M1 ipRGC lacking melanopsin.

**Figure 2:**
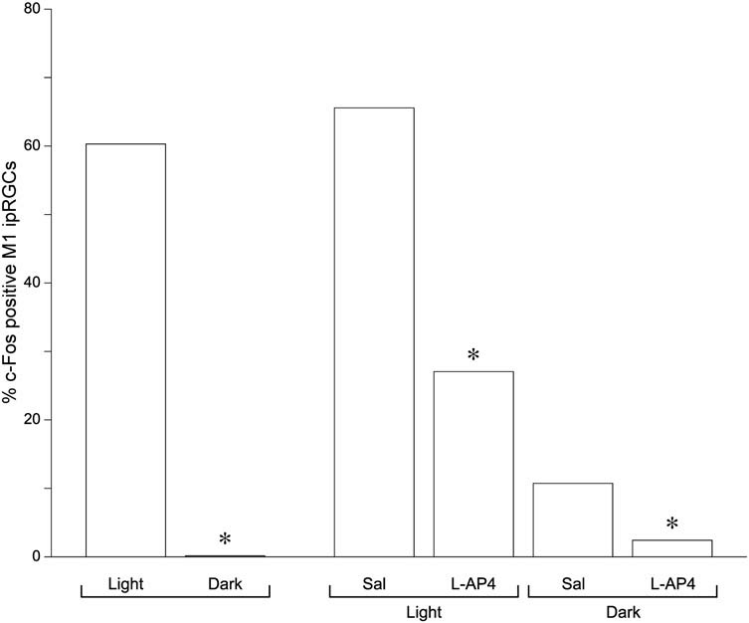
Light-induced c-Fos expression in ipRGCs lacking melanopsin. The percentage of β-galactosidase-positive ipRGCs expressing Fos in the retinas of *tau-lacZ^+/+^* mice three hr after light onset or at the same time of day but in the dark is shown on the far left of the histogram (white light = 1000 lux; n = 3 animals; dark = 0 lux; n = 2 animals) (* = p<0.0001, Fisher's exact test). The percentage of β-galactosidase-positive ipRGCs expressing Fos in the retinas of *tau-lacZ^+/+^* mice receiving an intravitreal injection of saline (sal) in one eye and L-AP4 in the other eye under dim red light conditions and killed after 3 h exposure to room light (white light = 1000 lux; n = 3 animals) or after 3 h exposure to complete darkness (dark = 0 lux; n = 3 animals) is provided on the right side of the histogram (* = p<0.0001, Fisher's exact test, sal vs L-AP4).

Experiments were conducted initially to determine if rod/cone photoreceptor driven input alone was capable of inducing Fos expression in ipRGCs lacking melanopsin. To this end, *tau-lacZ^+/+^* animals maintained in a 12:12 light:dark cycle were killed three hours after light-onset (n = 3) and the retinas were examined for Fos expression in β-galactosidase positive ganglion cells using a double-label immunocytochemical procedure incorporating chicken anti-β-galactosidase and rabbit anti-Fos primary antibodies. Animals serving as dark controls (n = 2) were killed at the same time of day except lights were kept off that day. Fos immunolabeling was detected in the ganglion cell layer of each animal killed in the light (Fig. 1) and the majority of β-galactosidase ganglion cells were Fos positive (60.3%; 363 Fos+ β-gal/602 β-gal) whereas virtually no Fos immunoreactivity was observed in the retinas of animals killed in the dark; only a single β-galactosidase ganglion cell was noted to be Fos-positive among the sections examined (0.1%; 1 Fos+ β-gal/713 β-gal) (Table 1; Fig. 2). These results establish that photic input via rod/cone signals alone is capable of inducing Fos expression in M1 ipRGCs lacking melanopsin.

**Table 1:** Light-induced Fos expression in ipRGCs.

Condition	Wild-type (%)*	Rd1 (%)*	tau-lacZ^+/+^ (%)**
Light	568/790 (71.9)	595/793 (75.0)	363/602 (60.3)
Dark	0/587 (0.0)	5/615 (0.8)	1/713 (0.1)

* Fos expression in melanopsin ipRGCs.

** Fos expression in β-galactoside ipRGCs. n = 3 animals for each group except Dark tau-lacZ^+/+^ where n = 2 animals.

Animals were killed three h after light onset or at the same time but in the dark. The number of Fos-positive melanopsin* or Fos-positive β-galactosidase** cells is presented as a percentage of the number of melanopsin or β-galactosidase cells. n = 3 animals for each group except for Dark tau-lacZ^+/+^ where n = 2.

Although these initial experiments validated the utility of using light-induced Fos expression to explore functional synaptic input to M1 ipRGCs, the question remained why only 60% of these cells showed Fos induction after light stimulation. Therefore we evaluated light-induced Fos expression in wild-type mice under conditions identical to those used for the *tau-lacZ^+/+^* animals except that a double-label immunocytochemical procedure employing rabbit primary antibodies for both melanopsin and Fos were used [14]. In the retinas of the wild-type animals (n = 3)≈72% of melanopsin ipRGCs expressed Fos in response to light stimulation (568 Fos+ mel/790 mel) (Table 1). In animals killed at the same time of day while maintained in the dark (n = 3), none of the 587 melanopsin ipRGCs examined in the wild-type mice were noted to be Fos-positive (Table 1), reaffirming the lack of Fos expression in the inner retina in the absence of light. Thus the combination of rod/cone-driven input plus the intrinsic melanopsin response to light induced Fos expression in the majority of melanopsin ipRGCs but clearly not in all of these inner retina photoreceptive neurons.

These results led us to question whether the synaptically driven and intrinsic responses to light were redundant in regard to light-induced Fos expression. Additional experiments were conducted examining light-induced Fos expression in rod/cone degenerate mice (mice homozygous for the *rd1* photoreceptor degeneration mutation; [31]) to provide an estimate of the extent to which light-induced Fos expression is a result of the intrinsic photosensitivity of these neurons. Under conditions similar to those used for the *tau-lacZ^+/+^* and wild-type animals, light stimulation induced Fos expression in 75% of melanopsin ipRGCs examined in the retinas of *rd1* animals (n = 3) (595 Fos+mel/793 mel), similar in magnitude to the light-induced Fos expression in melanopsin ipRGCs in wild-type animals (p>0.17, Fishers exact test; Table 1). In animals killed in the dark at the same time of day (n = 3) virtually no melanopsin ipRGCs were Fos immunopositive (0.8%; 5 Fos + mel/615 mel; Table 1). These results suggest a functional redundancy in extrinsic and intrinsic *c-fos* response to light stimulation.

The proportion of β-galactosidase positive ipRGCs expressing Fos following 3 h of light-stimulation in the *tau-lacZ^+/+^* animals (60%) may appear to be less than the proportion of melanopsin ipRGCs expressing Fos in wild-type and retinal degenerate mice under similar conditions (72–75%). However, a direct comparison between the *tau-lacZ^+/+^* and wild-type and *rd1* animals is not possible because in the *tau-lacZ^+/+^* mice only about one half of the melanopsin ipRGC population expresses β-galactosidase and therefore half of the ipRGCs could not be included in the analysis of their light responsiveness.

### ON-bipolar input to β-galactosidase ipRGCs in *tau-lacZ^+/+^* mice

To determine if light-induced Fos expression in M1 β-galactosidase ipRGCs is dependent on the retinal ON-bipolar functional circuit, *tau-lacZ^+/+^* animals were treated with L-AP4 prior to light stimulation. Under isofluorane anesthesia, animals received a unilateral intraocular injection of L-AP4 (1 µl; 100 µM final concentration) under dim red light (approximately 5 lux); the contralateral eye served as a control and was injected with 1 µl of vehicle (0.9% sterile saline) under the same dim red light conditions. After eyes were injected, anesthesia was terminated and five minutes after fully recovering from the anesthesia, animals (n = 3) were exposed to room light (1000 lux) for 3 h and were then killed. L-AP4 inhibition of the light responses of ON-bipolar cells is maintained for up to 4 h after intravitreal injection [32].

In the retinas from the saline injected eyes, light-induced Fos was detected in approximately 66% of the β-galactosidase ganglion cells (615 Fos+ β-gal/938 β-gal), very similar to number of Fos-positive β-galactosidase ganglion cells observed in animals killed 3 hr after light-onset (65.6% vs 60.3%). In the light-stimulated retinas from the L-AP4 injected eyes, 27% of the β-galactosidase ganglion cells were Fos-positive (295 Fos+ β-gal/1091 β-gal), a significant reduction compared to the saline injected eyes (27.0% L-AP4 vs 65.6% saline; p<0.0001, Fishers exact test; Table 2) (Fig. 2). These results demonstrate that inhibition of the ON-bipolar channel inhibits light-induced Fos expression in M1 ipRGCs lacking melanopsin.

**Table 2:** Intraocular application of L-AP4 inhibits light-induced Fos expression in M1 ipRGCs lacking melanopsin.

Condition	Animal No.	Treatment	Fos β-gal/β-gal (%)
Light	6	Sal	95/129 (73.6)
		L-AP4	91/343 (26.5)
	7	Sal	233/379 (61.5)
		L-AP4	62/311 (19.9)
	8	Sal	281/430 (65.3)
		L-AP4	142/437 (32.5)
Dark	9	Sal	21/86 (24.4)
		L-AP4	3/129 (2.3)
	10	Sal	20/184 (10.9)
		L-AP4	6/223 (2.7)
	11	Sal	11/215 (5.1)
		L-AP4	4/187 (2.1)

The number of Fos-positive β-galactosidase cells is presented as a percentage of the number of β-galactosidase cells observed for each eye of each animal (#1–#3) after intravitreal injection of 1 µl saline (sal) or 1 µl L-AP4 (1 mM) followed by 3 h of light stimulation or 3 h of darkness.

Dark control animals (n = 3) received L-AP4 in one eye and saline in the other eye under similar dim red light conditions, but after recovering from anesthesia the animals were returned to the dark and killed 3 h later. In the retinas from the saline injected eyes, a small number of β-galactosidase-positive ganglion cells were noted to be Fos-positive (10.7%; 52 Fos+ β-gal/485 β-gal). The number of Fos-positive β-galactosidase ganglion cells was reduced to almost zero in the retinas of the L-AP4 injected eyes (2.4%; 13 Fos+ β-gal/539 β-gal) (Fig. 2). Since no Fos was detected in β-galactosidase ganglion cells in the uninjected *tau-lacZ^+/+^* animals killed in complete darkness, the low level of Fos expression in ipRGCs in the animals that received intravitreal injections is interpreted to be due to a combination of the physical insult of the intravitreal injection and to some extent from the red light exposure the animals received while the eyes were being injected, since L-AP4 was effective in reducing Fos expression within this dark control population. Taken together the results indicate that light can induce Fos expression in ipRGCs via a synaptically driven mechanism or via phototransduction mediated by the melanopsin photopigment and that the ON-bipolar channel provides input to ipRGCs with dendrites restricted to the OFF substratum of the IPL.

## Discussion

The principal novel finding in this study is that intrinsically photosensitive retinal ganglion cells in *tau-lacZ^+/+^* mice that lack melanopsin photopigment (*opn4^−/−^*) express the immediate early gene *c-fos* following light stimulation. In *tau-lacZ^+/+^* mice a subset of ipRGCs is identified by their expression of β-galactosidase (M1 ipRGCs) and these neurons send dendrites to the OFF substrata of the IPL where they stratify near the border of the inner nuclear layer (INL) [14]. The observation of light-induced Fos expression in β-galactosidase M1 ipRGCs *in vivo* confirms a previous report using an *in vitro* preparation and intracellular recording from the primate retina indicating that ipRGCs with dendrites in the OFF sublamina of the IPL receive photoreceptor input via cone ON-bipolar cells [8]. The present results extend those previous findings by demonstrating that the light evoked signals driving Fos expression *in vivo* are inhibited by the potent metabotropic glutamate agonist L-AP4, which selectively blocks transmission of signals between photoreceptors and ON-bipolar cells by acting as a substitute for photoreceptor released glutamate at the mGluR6 receptor [30], [33].

It has been reported previously using whole-cell patch recording of ipRGCs in the rat retina maintained *in vitro*, that L-AP4 abolishes ON depolarization in ipRGCs [10]. It was suggested that these results provided evidence for ON-bipolar input to OFF-stratifying ganglion cells [10]. However, there are multiple types of ipRGC in the rat, with some cells sending dendrites into the distal IPL and others distributing dendrites to the proximal IPL [3], similar to ipRGCs in mouse [7], [11], [12], [14]] and primate retinas [8], [34]. Since the ganglion cells recorded by Wong and colleagues [10] that were inhibited by L-AP4 were not filled after recording, it is not known whether these ganglion cells had dendrites in the ON or OFF substrata of the IPL. However, it is likely that Wong and colleagues [10] recorded from a population of ipRGCs in the rat retina that was biased for OFF-stratifying ipRGCs; ganglion cells targeted for *in vitro* recording were identified following retrograde transport of tracer injected into the hypothalamus *in vivo*. In the mouse retina, 80% of ipRGCs projecting to the hypothalamus are M1 OFF-stratifying ipRGCs [14].

The correlation between the level at which a bipolar axon arborizes in the inner plexiform layer and its physiological response to light is very strong; ON-bipolars arborize in the inner half of the IPL whereas OFF-bipolars arborize in the outer half of the IPL. This reflects a fundamental segregation of neural interconnections of the inner retina based on a distinction made in the outer retina that appears to apply to all mammalian species [35]. Thus, the demonstration of an ON-bipolar input to a ganglion cell with dendrites in the outer OFF layer of IPL as described in the current study in a rodent and by Dacey and colleagues [8] in a primate is very unusual. Although our data strongly implicate the ON-bipolar circuit in the light-evoked expression of Fos in M1 ipRGCs, the structural basis for this unusual circuit is currently unknown and the pathway could be either direct or indirect.

**Figure 3:**
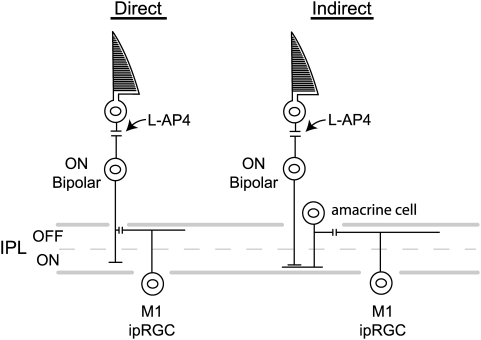
ON-bipolar cell input to M1 ipRGCs may be either direct or indirect. L-AP4 acts at the mGluR6 receptor located on the dendrites of ON-bipolar cells. L-AP4 inhibition of light-induced Fos expression in M1 ipRGCs establishes that this functional channel provides synaptic drive to M1 ipRGCs. However, the results do not differentiate between a direct pathway of ON-bipolar input to M1 ipRGC dendrites in the outer OFF sublamina of the IPL vs an indirect circuit via a potential intervening glutamatergic amacrine cell.

A potential indirect ON-bipolar pathway to ipRGCs with dendrites in the OFF substratum of the IPL could arise from ON-bipolar cell input to a small subset of amacrine cells that are glutamatergic with processes in the ON sublamina of IPL [36], [37]. These glutamatergic amacrine cells would then need to send processes to synapse on ipRGC dendrites in the OFF sublayer of the IPL (Fig. 3). Although glutamatergic amacrine cells have been described in the INL of the rodent retina, the processes of these amacrine cells descend to form a broad plexus in the mid-IPL [36], [37] and thus would seem to be ill-suited to convey ON-bipolar signals to the dendritic arbors of M1 ipRGCs in the distal IPL near the INL.

A direct ON-bipolar input to ipRGCs with dendrites in the outer IPL seems more likely. On rare occasion smooth axons of mammalian bipolar cells have been described to possess collateral offshoots [38]. More recently, using a transgenic mouse line (*Grm6-GFP*) in which ON-bipolar cells express green fluorescent protein (GFP) under the control of the promoter of the gene *Grm6*, which encodes mGluR6 [39], Wong et al. [40] have provided a preliminary report using light microscopic techniques in which they describe ectopic terminal-like structures of ON-bipolars in the OFF substratum of the IPL. These ectopic bipolar synapses were noted to be in close apposition to dendrites of melanopsin ipRGCs near the IPL-INL border [40]. These data are consistent with our current findings in the mouse and support the direct pathway of ON-bipolar input to ipRGCs with dendrites in the OFF substrata of the IPL. Electron microscopic confirmation establishing that the ON-bipolars are truly presynaptic to the ipRGCs with dendrites in the outer stratum of IPL would further strengthen this conclusion.

The functional evidence provided in the current report for cone ON-bipolar input to ipRGCs stratifying in the OFF layer of the IPL does not address the possibility that cone ON-bipolar cells may make synaptic contacts with the very small portion of the dendritic arbor of M1 ipRGCs that traverses the proximal ON sublayer of the IPL before branching in the OFF substratum near the INL. Nor does it differentiate the possibility of rod ON-bipolar cell input directly to the ipRGC soma as suggested by Østergaard et al. [6]. However, analyses of the receptive field for synaptically mediated depolarizations in ipRGCs resembles that of the dendritic field of these cells suggesting that ipRGCs receive cone ON-bipolar inputs throughout their entire dendritic fields [10]. If cone ON-bipolar synaptic contacts were limited to the dendritic arbor of M1 ipRGCs in the ON sublayer of the IPL or rod ON-bipolar inputs were limited to the soma, the receptive fields of ipRGCs would be expected to be considerably smaller than the dendritic fields. The available data do not eliminate the possibility of rod and cone ON-bipolar inputs to soma and proximal dendrites of ipRGCs but they do suggest that M1 ipRGCs receive considerable cone ON-bipolar synaptic input to their distal dendrites in the OFF sublamina.

The interpretation that light-induced Fos expression in ipRGCs is completely driven by rod/cone photoreceptor mediated synaptic input in the *tau-lacZ^+/+^* mouse is dependent on the lack of an intrinsic melanopsin-mediated response to light in these cells. Immunocytochemical analyses have revealed no melanopsin immunostaining in the retinas of the *tau-lacZ^+/+^* mouse [13], [14]. In addition, Lucas and colleagues [29] recorded from RGCs in the *tau-lacZ^+/+^* mouse that were retrogradely labeled after tracer injection into the hypothalamic suprachiasmatic nucleus (SCN), a known target of melanopsin ipRGCs [2], [41]. In the presence of 2 mM CoCl_2_ to block synaptic transmission, none of the neurons recorded were photosensitive [29]. We conclude therefore that the light-induced Fos expression observed in 60–66% of the β-galactosidase RGCs in this current study was synaptically driven and independent of an intrinsic light response. This proportion of ipRGCs expressing Fos is in the range of light-induced Fos expression observed in wild-type mice and in mice lacking rods and cones (70–75%, this study, [42]). These data may suggest a high level of redundancy between the rod/cone excitatory drive to M1 ipRGCs and the endogenous melanopsin-mediated response to light in driving Fos expression. This seems reasonable since both the light-evoked extrinsic input and the intrinsic photo response both produce sustained depolarizations of the ipRGCs [4], [10]. The redundancy suggested here stands in contrast to the interpretation of Semo et al. [42] that “most of the light-induced *c-fos* expression within these cells is associated with the endogenous photosensitivity of these neurons” although these authors did not examine synaptically driven Fos expression in their study.

Mammalian ipRGCs share several features with classical retinal ganglion cells; they send dendrites into the IPL where they are contacted by bipolar and amacrine cells. They utilize glutamate as a neurotransmitter and they send axons to retinorecipient structures in the brain. However, these novel ganglion cells may be more different than they are similar to classical ganglion cells. They express a photopigment melanopsin [43] and they apparently utilize a phototransduction mechanism that more closely resembles that of invertebrates [4]. Melanopsin ipRGCs also provide excitatory drive to dopaminergic amacrine neurons, establishing that information flow from these cells is bi-directional [28]. As we have now demonstrated that they do not conform to the fundamental segregation of ON and OFF channels in the vertebrate retina, it seems almost certain that we have just begun to reveal the idiosyncrasies of these peculiar ganglion cells.

## Methods

### Animals

Mice of either sex of a mixed B6/129 strain (10–16 weeks old) genetically modified to generate a tau-lacZ protein in place of the melanopsin protein (Hattar et al., 2002) were used in this study as well as 15–16 week old wild-type (C57BL/6J) and C3H/HeJ mice (homozygous for the retinal degeneration allele Pde6b^rd1^). C57BL/6J and C3H/HeJ were obtained from Jackson laboratory, Bar Harbor, ME). The *tau-lacZ* knock-in mice were raised in our laboratory from mice generously provided by Dr. Samer Hattar (Johns Hopkins University). Animals were maintained under a 12:12 light:dark cycle, with lights on at 08.00h and food and water were available *ad libitum*. All animals were killed at 11.00h. Experiments were performed according to the National Institutes of Health Guidelines for the Care and Use of Laboratory Animals, and were approved by the Colorado State University Animal Care and Use Committee.

### Intraocular injections

Mice were anesthetized by isofluorane (2.5–5%) inhalation anesthesia. Under dim red light (5 lux) provided by a Kodak safelight (GBX-2 filter) equipped with a 7.5 watt bulb, 1 µl of 1 mM L-AP4 (L-(+)-2-amino-4-phosphonobutyric acid; Tocris, Ellisville, MO) in sterile 0.9% saline was injected into the vitreous body of one eye and 1 µl sterile 0.9% saline was injected into the vitreous body of the other eye using a glass micropipette attached to a Nanoject II (Drummond Scientific, Broomall, PA). After recovering from anesthesia, animals were either exposed to bright room light (1000 lux) or were maintained in complete darkness. In preparation for immunocytochemical analysis, animals were anesthetized with sodium pentobarbital (80 mg/kg i.p.) and perfused transcardially with 0.9% saline followed by cold freshly prepared fixative consisting of 4% paraformaldehyde in 0.1 M phosphate buffer (pH 7.3). Eyes were enucleated and the anterior segments and vitreous were removed. The eyecups were embedded in 7% gelatin (100 bloom, Fisher Scientific) and placed in the same fixative with 20% sucrose overnight at 4°C. Eyecups were sectioned at 40 µm on a sliding microtome equipped with a freezing stage (Physitemp Instruments, Clifton, NJ) and sections were collected in phosphate-buffered saline (PBS).

### Immunocytochemistry

Double-label light microscopic immunocytochemistry was performed as described previously [14], [28]. Briefly, for Fos and β-galactosidase immunolabeling, free-floating sections were incubated in chicken anti-β-galactosidase antibody diluted 1∶500 (ab9361, Abcam, Cambridge, MA) and rabbit anti-Fos antibody diluted 1∶10000 (AB-5 Calbiochem) followed by goat anti-rabbit IgG conjugated to Alexa Fluor 488 and goat anti-chicken IgY conjugated to Alexa Fluor 594 (Molecular Probes). Double-label immunocytochemistry for Fos and melanopsin utilized two rabbit primary antibodies and was conducted serially as described previously [14]. Briefly, sections were initially processed for Fos as described above and after rinsing were incubated in rabbit anti-lucifer yellow (1∶200; any rabbit IgG generated against an antigen not found in the native tissue can be used at this step) and after further rinsing were blocked in donkey anti-rabbit Fab (Jackson Immunoresearch, West Grove, PA), rinsed and incubated in rabbit anti-melanopsin (UF006 generously provided by Ignacio Provencio, University of Virginia) directly conjugated to Alexa Fluor 594 using a Zenon kit as described by the manufacturer (Molecular Probes). Sections were rinsed followed by incubation in 4% paraformaldehyde in PBS, washed, mounted on subbed slides, coverslipped with Vectashield and sealed. Sections were examined using a Leica (Nussloch, Germany) DMRA light microscope and images were captured using a Hamamatsu (Hamamatsu City, Japan) C4742-95 CCD digital camera and deconvolved using Openlab fluorescence deconvolution software (Improvision, Boston, MA). Digital images were pseudo-colored, and images were prepared using Adobe Photoshop version 6.0.1; images were enhanced for brightness and/or contrast. All β-galactosidase and melanopsin immunopositive cells were counted in the retinal sections examined and the number of β-galactosidase and melanopsin ganglion cells expressing Fos was determined.

## References

[1] Berson DM, Dunn FA, Takao M (2002). Phototransduction by retinal ganglion cells that set the circadian clock.. Science.

[2] Hattar S, Liao HW, Takao M, Berson DM, Yau KW (2002). Melanopsin-containing retinal ganglion cells: architecture, projections, and intrinsic photosensitivity.. Science.

[3] Warren EJ, Allen CN, Brown RL, Robinson DW (2003). Intrinsic light responses of retinal ganglion cells projecting to the circadian system.. Eur J Neurosci.

[4] Hartwick ATE, Bramley JR, Yu J, Stevens KT, Allen CN (2007). Light-evoked calcium responses of isolated melanopsin-expressing retinal ganglion cells.. J Neurosci.

[5] Belenky MA, Smeraski CA, Provencio I, Sollars PJ, Pickard GE (2003). Melanopsin retinal ganglion cells receive bipolar and amacrine cell synapses.. J Comp Neurol.

[6] Østergaard J, Hannibal J, Fahrenkrug J (2007). Synaptic contact between melanopsin-containing retinal ganglion cells and rod bipolar cells.. Invest Ophthal Vis Sci.

[7] Viney TJ, Balint K, Hillier D, Siegert S, Boldogkoi Z (2007). Local retinal circuits of melanopsin-containing ganglion cells identified by transsynaptic viral tracing.. Curr Biol.

[8] Dacey DM, Liao HW, Peterson BB, Robinson FR, Smith VC (2005). Melanopsin-expressing ganglion cells in primate retina signal colour and irradiance and project to the LGN.. Nature.

[9] Perez-Leon JA, Warren EJ, Allen CN, Robinson DW, Brown RL (2006). Synaptic inputs to retinal ganglion cells that set the circadian clock.. Eur J Neurosci.

[10] Wong KY, Dunn FA, Graham DM, Berson DM (2007). Synaptic influences on rat ganglion-cell photoreceptors.. J Physiol.

[11] Schmidt TM, Taniguchi K, Kofuji P (2008). Intrinsic and extrinsic light responses in melanopsin-expressing ganglion cells during mouse development.. J Neurophysiol.

[12] Schmidt TM, Kofuji P (2009). Functional and morphological differences among intrinsically photosensitive retinal ganglion cells.. J Neurosci.

[13] Hattar S, Kumar M, Park A, Tong P, Tung J (2006). Central projections of melanopsin-expressing retinal ganglion cells in the mouse.. J Comp Neurol.

[14] Baver SB, Pickard GE, Sollars PJ, Pickard GE (2008). Two types of melanopsin retinal ganglion cell differentially innervate the hypothalamic suprachiasmatic nucleus and the olivary pretectal nucleus.. Eur J Neurosci.

[15] Snellman J, Kaur T, Shen Y, Nawy S (2008). Regulation of ON bipolar cell activity.. Prog Retinal Eye Res.

[16] Werblin FS, Dowling JE (1969). Organization of the retina of the mudpuppy, Necturus maculosus. II. Intracellular recording.. J Neurophysiol.

[17] Kaneko A (1970). Physiological and morphological identification of horizontal, bipolar and amacrine cells in goldfish retina.. J Physiol.

[18] Numura A, Shigemoto R, Nakamura Y, Okomoto N, Mizumo N (1994). Developmentally-regulated postsynaptic localization of a metabotropic glutamate receptor in rat rod bipolar cells.. Cell.

[19] Vardi N, Morigawa K (1997). ON cone bipolar cells in rat express the metabotropic receptor mGluR6.. Vis Neurosci.

[20] Gilbertson TA, Scobey R, Wilson M (1991). Permeation of calcium ions through non-NMDA glutamate channels in retinal bipolar cells.. Science.

[21] Famiglietti EV, Kolb H (1976). Structural basis for ON- and OFF-center responses in retinal ganglion cells.. Science.

[22] Sagar SM, Sharp FR (1990). Light induces a Fos-like nuclear antigen in retinal neurons.. Mol Brain Res.

[23] Chambille I, Doyle S, Serviere J (1993). Photic induction and circadian expression of Fos-like protein. Immunohistochemical study in the retina and suprachiasmatic nuclei of hamster.. Brain Res.

[24] Koistinaho J, Sagar SM (1995). Light-induced c-fos expression in amacrine cells in the rabbit retina.. Mol Brain Res.

[25] Rohrer B, Iuvone PM, Stell WK (1995). Stimulation of dopaminergic amacrine cells by stroboscopic illumination of fibroblast growth factor (bFGF, FGF-2) injections: possible roles in prevention of form deprivation myopia in the chick.. Brain Res.

[26] Huerta JJ, Llamosas MM, Cernuda-Cernuda R, Garcia-Fernandez JM (1997). Fos expression in the retina of rd/rd mice during the light/dark cycle.. Neurosci Lett.

[27] Hanzlicek BW, Peachey NS, Grimm C, Hagstrom SA, Ball SL (2004). Probing inner retinal circuits in the rod pathway: a comparison of c-fos activation in mutant mice.. Vis Neurosci.

[28] Zhang D-Q, Wong KY, Sollars PJ, Berson DM, Pickard GE (2008). Intraretinal signaling by ganglion cell photoreceptors to dopaminergic amacrine neurons.. Proc Natl Acad Sci USA.

[29] Lucas RJ, Hattar S, Takao M, Berson DM, Foster RG (2003). Diminished pupillary light reflex at high irradiances in melanopsin-knockout mice.. Science.

[30] Slaughter MM, Miller RF (1981). 2-amino-4-phosphonobutyric acid: a new pharmacological tool for retina research.. Science.

[31] Bowes C, Li T, Danciger M, Baxter LC, Applebury ML, Farber DB (1990). Retinal degeneration in the rd mouse is caused by a defect in the beta subunit of rod cGMP-phosphodiesterease.. Nature.

[32] Dolan RP, Schiller PH (1994). Effects of ON channel blockade with 2-amino-4-phosphonobutyrate (APB) on brightness and contrast perception in monkeys.. Vis Neurosci.

[33] Nakajima Y, Iwakabe H, Akazawa C, Nawa H, Shigemoto R (1993). Molecular characterization of a novel retinal metabotropic glutamate receptor mGluR6 with a high agonist selectivity for L-2-amino-4-phosphonobutyrate.. J Biol Chem.

[34] Jusuf PR, Lee SC, Hannibal J, Grunert U (2007). Characterization and synaptic connectivity of melanopsin-containing ganglion cells in the primate retina.. Eur J Neurosci.

[35] Dowling JE (1987). The Retina: An approachable Part of the Brain.

[36] Johnson J, Sherry DM, Liu X, Fremeau RT, Seal RP (2004). Vesicular glutamate transporter 3 expression identifies glutamatergic amacrine cells in the rodent retina.. J Comp Neurol.

[37] Haverkamp S, Wassle H (2004). Characterization of an amacrine cell type of the mammalian retina immunoreactive for vesicular glutamate transporter 3.. J Comp Neurol.

[38] Rodieck RW (1973). The Vertebrate Retina: Principles of Structure and Function.

[39] Morgan JL, Dhingra A, Vardi N, Wong ROL (2006). Axons and dendrites originate from neuroepithelial-like processes of retinal bipolar cells.. Nat Neurosci.

[40] Wong KY, Dumitrescu ON, Pucci FG, Berson DM (2008). Paradoxical ON bipolar cell input to the OFF sublamina of the inner plexiform layer provides a substrate for ON channel input to ganglion-cell photoreceptors.. Soc Neurosci.

[41] Sollars PJ, Smeraski CA, Kaufman JD, Ogilvie MD, Provencio I (2003). Melanopsin and non-melanopsin expressing retinal ganglion cells innervate the hypothalamic suprachiasmatic nucleus.. Vis Neurosci.

[42] Semo M, Lupi D, Peirson SN, Butler JN, Foster RG (2003). Light-induced *c-fos* in melanopsin retinal ganglion cells of young and aged rodless/coneless (*rd/rd c*l) mice.. Eur J Neurosci.

[43] Provencio I, Rollag MD, Castrucci AM (2002). Photoreceptive net in the mammalian retina.. Nature.

